# Single Donor FMT Reverses Microbial/Immune Dysbiosis and Induces Clinical Remission in a Rat Model of Acute Colitis

**DOI:** 10.3390/pathogens10020152

**Published:** 2021-02-02

**Authors:** Petra Adamkova, Petra Hradicka, Sona Gancarcikova, Monika Kassayova, Lubos Ambro, Izabela Bertkova, Martin Maronek, Silvia Farkasova Iannaccone, Vlasta Demeckova

**Affiliations:** 1 Institute of Biology and Ecology, Faculty of Science, Pavol Jozef Safarik University in Kosice, 041 54 Kosice, Slovakia; petra.adamkova@student.upjs.sk (P.A.); p.hradicka@gmail.com (P.H.); monika.kassayova@upjs.sk (M.K.); 2 Department of Microbiology and Immunology, University of Veterinary Medicine and Pharmacy in Kosice, 041 81 Kosice, Slovakia; sona.gancarcikova@uvlf.sk; 3 Department of Experimental Medicine, Faculty of Medicine, Pavol Jozef Safarik University in Kosice, 040 11 Kosice, Slovakia; lubos.ambro@upjs.sk (L.A.); izabela.bertkova@upjs.sk (I.B.); 4 Institute of Molecular Biomedicine, Faculty of Medicine, Comenius University in Bratislava, 811 08 Bratislava, Slovakia; martin.maronek@gmail.com; 5 Department of Forensic Medicine, Faculty of Medicine, Pavol Jozef Safarik University in Kosice, 040 11 Kosice, Slovakia; silvia.farkasova.iannaccone@upjs.sk

**Keywords:** DSS-induced colitis, faecal microbial transplant, ulcerative colitis, IBD, cytokines

## Abstract

Deviation in the gut microbial composition is involved in various pathologies, including inflammatory bowel disease (IBD). Faecal microbiota transplant (FMT) can act as a promising approach to treat IBD by which changes in microbiome can be reversed and homeostasis restored. Therefore, the aim of this study was to investigate the effect of FMT on the remission of acute inflammatory response using dextran sulfate sodium (DSS)-induced rat colitis model. Faecal microbial communities were analysed using the 16S rRNA approach, and clinical manifestations together with histological/haematological/biochemical/immunological analyses were assessed. Our study demonstrated significant shifts in the dominant species of microbiota under inflammatory conditions induced by DSS and evident restoration effect of FMT treatment on microbial composition. These faecal microbial alterations in FMT-treated rats led to a relative restoration of colon length, and a significant decrease in both epithelium damage and disease severity, which was reflected in lower serum pro-inflammatory cytokine levels. Haematological/biochemical parameters in DSS-treated animals showed signs of anaemia with a significant reduction in red blood cell count together with increasing levels of total bilirubin, creatinine and phosphorus suggesting potential protective effect of FMT. These results support FMT as a valuable therapeutic strategy to control inflammation during acute colitis.

## 1. Introduction

Inflammatory bowel disease (IBD) is a chronic, multifactorial inflammation of the gastrointestinal tract (GIT), mainly represented by Crohn’s disease (CD) and ulcerative colitis (UC) and characterised by a relapsing clinical course, requirement for lifelong medication and, often, significant morbidity. Recent epidemiological data suggest that the incidence and prevalence of the diseases are increasing and that an estimated 2.5–3 million people in Europe are affected by IBD with a direct healthcare cost of 4.6–5.6 billion Euros/year [1]. Moreover, the occurrence of IBD is a dynamic process as increasing incidence rates are being reported from previously low incidence areas such as Eastern Europe [2,3]. While multiple effective therapeutic options exist for the treatment of IBD, a significant proportion of patients will either fail to respond or lose response to the therapy. Early treatment options include corticosteroids, followed by immune modulators and monoclonal antibodies [4]. The fact that many patients with IBD do not respond adequately to existing therapies suggests that there are still unknown aspects to the mechanism of IBD. In addition, many of these IBD medications were associated with significant infectious and neoplastic side effects [5,6]. Therefore, it is clear that the development and implementation of new highly effective treatments or drug combinations with less toxic side-effects is an important research priority. In fact, the management of IBD has changed significantly in the past two decades based on a detailed understanding of the inflammatory pathways that are activated in IBD. During this period, the medical field has witnessed the development of treatment agents that specifically target inflammatory processes in the gut [7]. Virtually all currently approved and most often prescribed treatments for IBDs are directed toward the over-active immune response rather than the intestinal bacteria. However, during the last decade, it has been recognised that “dysbiosis”, an imbalanced gut microbiota, represents another key element in IBD [8]. Microbiota dysbiosis in patients with IBD is associated with reduced diversity of microorganisms, due to a reduced incidence of beneficial bacterial species and/or their replacement by pathogens. Compared to healthy individuals, a decrease in bacteria with anti-inflammatory effects and an expansion of pro-inflammatory bacteria have been observed in patients with IBD [9]. In this context, therapies aimed at modulating/restoring the gut microbiota to a premorbid state have been suggested as one of the most promising strategies to treat immune-mediated diseases [10]. Currently, a wide variety of therapeutic strategies are used to reverse gut dysbiosis, but a great majority of them do not exert satisfactory clinical effects, except for treatment using faecal microbiota transplant (FMT). FMT treatment is the transfer of minimally manipulated, pre-screened donor stool into the patient’s GIT, with the aim of ameliorating the dysbiotic state by increasing overall diversity as well as restoring the functionality of the microbiota [11]. This therapeutic approach has been shown to be incredibly successful in the treatment of recurrent *Clostridium difficile* infection, however it is potentially beneficial in other microbiota-related disorders as well [12,13,14,15]. The underlying microbial basis, predictors of therapeutic outcome and the active constituents of FMT which mediate benefit remain unknown. However, this microbial-based therapy has demonstrated the ability to reduce both the dysbiotic environment and production of inflammatory mediators [16]. The selection of a suitable donor based on microbial indicators is a key point in FMT treatment, therefore strict screening tests are recommended to improve its efficacy and prevent the occurrence of side effects [17]. Moreover, FMT delivery methods and microbial strains need to be rationally designed and tightly controlled taking into account microbial diversity and taxonomic composition. FMT treatment in its current form is not a one size fits all strategy, and more studies are required to identify those microbial components of the faecal microbiota that have specific effects in patients with different diseases. To determine whether FMT therapy may play a main and/or complementary role in the standard treatment of IBD, further studies are needed to help develop a more efficient personalised approach for patients with IBD.

The present study aims to evaluate the modulatory and restorative effects of FMT therapy on inflammatory responses in a chemically induced rat colitis model.

## 2. Results

### 2.1. Dextran Sulfate Sodium (DSS)-Induced Rat Colitis

No mortality was observed among the experimental groups during the entire experimental period. As the most frequently detected symptom of IBD in the clinic is weight loss, the mean daily weight gain following DSS treatment was measured. All rats gained weight throughout the experimental period without statistical differences between experimental groups, but a trend in smaller weight gain in the DSS group is obvious despite a significantly higher food intake (*p* < 0.001) suggesting decreased ability to process food (Table 1). On the other hand, the lowest food intake in the FMT group was not reflected in their mean weight gain, indicating that the ability to convert food to body weight was positively affected by FMT. This observation was confirmed by significantly higher food efficiency ratio (FER) in the FMT group compared with the DSS group (*p* < 0.05). These results suggest an unaffected ability to convert food to body mass by FMT treatment in acute colitis and its positive effect on food digestion and absorption.

### 2.2. Microbial Composition of Faecal Transplant

**Figure 1:**
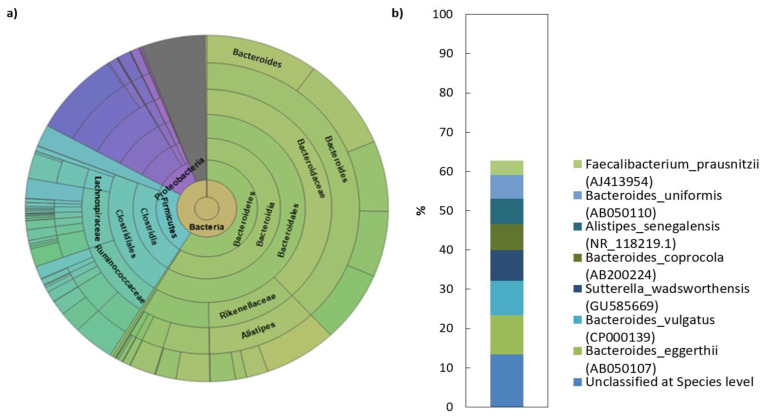
Microbial composition of human faecal transplant: (**a**) Krona diagram visually displays the result of taxonomic annotation analysis. Circles from inside to outside stand for different taxonomic ranks showing the bacterial diversity in the stool sample of donor transplant up to the taxonomic level of the family; (**b**) top species classification results: This diagram shows the top 8 species of 681 total species-level taxonomic categories identified.

The analysis of the sequencing data revealed that the human faecal transplant comprised of eight different phyla, of which 99.2% belong to Bacteroidetes, Firmicutes, Proteobacteria and Actinobacteria (Table 2). Among these, Bacteroidetes and Firmicutes dominated the microbiota in donor’s gut (>80% of the total number of bacteria). More complex taxonomy data are available in Figure 1. The Bacteroidetes phylum was mainly represented by members of the families Bacteroidaceae (38.5%) and Rikenellaceae (11.7%). The dominant genera were represented by *Bacteroides* (38%) and *Alistipes* (11.7%). In contrast to what was found with the Bacteroidetes, the phylum Firmicutes in donor sample was represented by a more diverse mix of families and was dominated by Lachnospiraceae, Ruminococcaceae and unclassified Firmicutes. Erysipelotrichaceae and unclassified Clostridiales were also present, but in lower abundance than the other families. NGS analysis of the donor transplant did not confirm the presence of pathogenic species.

### 2.3. Microbial Composition of Rat Faecal Samples

**Figure 2:**
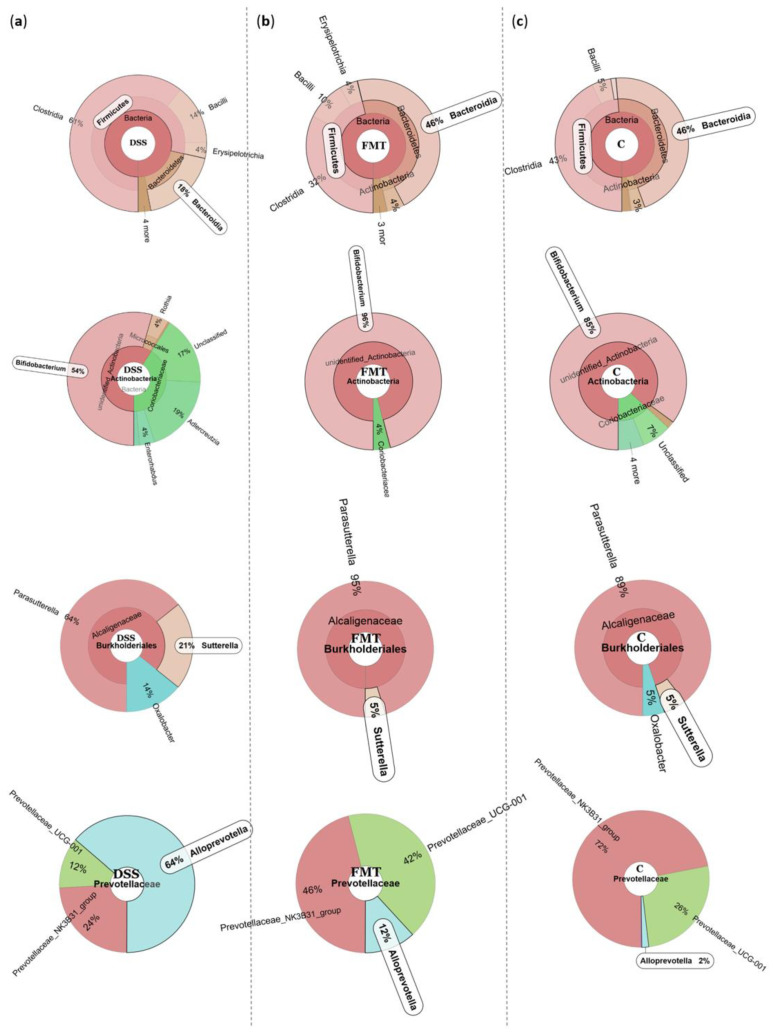
Representative Krona diagrams visually display the effects of DSS on relative abundance of gut microbiota in the experimental groups of rats. Circles from inside to outside stand for different taxonomic ranks: (**a**) DSS—dextran sulfate sodium induced colitis (positive control); (**b**) FMT—DSS-induced colitis with subsequent FMT treatment; (**c**) C—healthy control.

**Figure 3:**
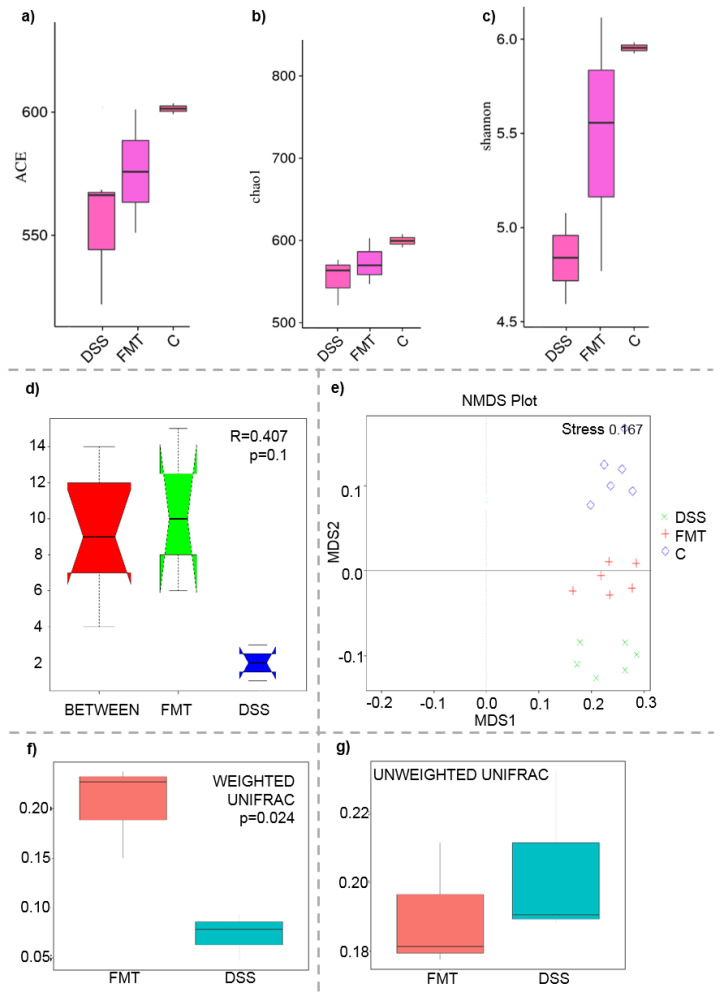
Boxplot of alpha diversity indices (**a**–**c**) and various beta diversity analysis (**d**–**g**): (**a**) ACE; (**b**) Chao1; (**c**) Shannon; (**d**) beta distance; (**e**) non-metric multidimensional scaling (NMDS) analysis; (**f**) weighted UniFrac beta diversity among FMT and DSS groups; (**g**) unweighted UniFrac beta diversity among FMT and DSS groups. Alpha diversity indexes are composite indexes reflecting abundance and consistency. Experimental groups were not significantly different by the ACE, Chao 1 and Shannon index measures of diversity. ACE and Chao1 indices reflect the OTU abundance in samples, Shannon indices reflect the diversity of OTU in samples. The greater the Chao or ACE index, the higher the expected species richness of the microbiota; the greater the Shannon index, the higher the diversity of the microbiota; (**d**) box plot of Inter-group and Intra-group Beta distance (ANOSIM Analysis). The *x*-axis represents the grouping and the *y*-axis represents the distance calculated by Unweighted_unifrac. The data in the box are the distance of Inter-group and Intra-group, respectively. R-value range (−1, 1). The R-value > 0 shows that inter-group differences are greater than intra-group differences. The *p*-value represents the confidence level of the statistical analysis; (**b**) non-metric multidimensional scaling (NMDS) analysis. Each point in the graph represents one sample, and different colours represent different groups. The distance between points represents the level of difference. Stress lower than 0.2 indicates that the NMDS analysis is reliable. The closer the samples in the graph, the higher their similarity. (**c**,**d**) Boxplots of weighted (a quantitative measure) and unweighted (qualitative measure) UniFrac beta diversity among FMT and DSS groups. Weighted UniFrac measure is well suited for detecting differences in abundance even when the overall groups of organisms that are present in each sample remain the same. Unweighted UniFrac measure is well suited for detecting differences in the presence or absence of lineages of bacteria in different communities.

**Figure 4:**
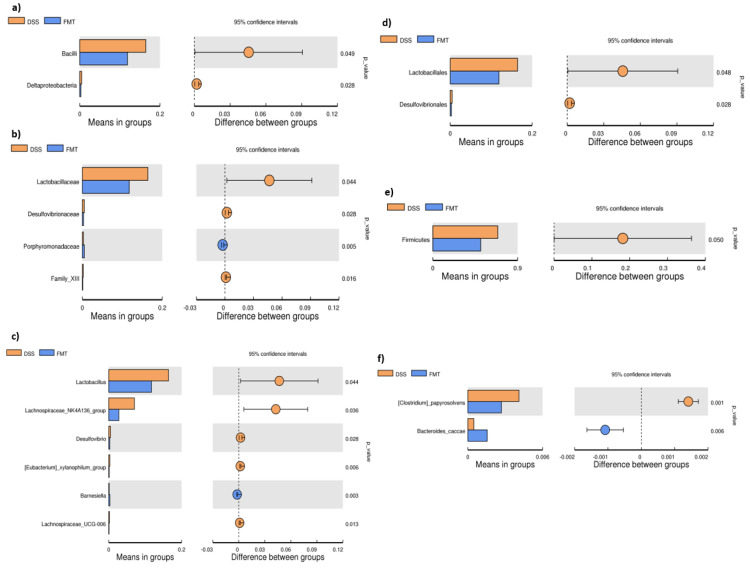
T-test bar plot of significantly different faecal microbial species in DSS and FMT groups at different taxonomic ranks: (**a**) Class; (**b**) family; (**c**) genus; (**d**) order; (**e**) phylum; (**f**) species. DSS—dextran sulfate sodium induced colitis (positive control); FMT—DSS-induced colitis with subsequent FMT treatment.

The microbiological changes observed in faeces of animals in experimental groups are shown in Figure 2, Figure 3 and Figure 4. A detailed phylogenetic analysis of the microbial taxonomic composition in the faeces of rats with induced colitis showed that the reduced inflammatory conditions observed upon FMT administration were associated with variations in the abundance of specific taxa, including Firmicutes and Bacteroidetes. Alpha-diversity indices (Ace, Chao 1 and Shannon) (Figure 3a–c) and beta-diversity indices (Figure 3d–g) in the faeces of DSS, FMT groups and healthy animals were calculated. The beta diversity analysis of weighted UniFrac metric showed a significant difference between DSS and FMT groups (*p* < 0.05). Treatment with 5% DSS for 7 days significantly increased abundance of Firmicutes (*p* < 0.05) and decreased abundance of Bacteroidetes and Actinobacteria in the DSS group compared to control and FMT-treated rats (Figure 2). Replacement of DSS with water for the duration of FMT treatment did not reverse these changes in the DSS group. Significant changes towards restoration of normobiosis were detected among the less abundant families belonging to the Firmicutes phylum in the FMT group. For instance, Clostridia, which were expanded in the DSS group, were reduced upon FMT treatment and returned to levels comparable to those observed in control rats (Figure 2). *Clostridium papyrosolvens* represented the bacterial population that was significantly increased in the DSS group (*p* < 0.001; Figure 4), together with significant decrease of *Bacteroides caccae* (*p* < 0.01). DSS administration also induced a decrease in *Bifidobacterium* and an increase in proportion of the family Coriobacteriaceae, which are major representatives of the phylum Actinobacteria. These microbial changes were also reversed in FMT-treated rats (Figure 2). Moreover, they showed lower abundance of the genus *Alloprevotella* (Bacteroidetes) as well as *Suterella* and *Desulfovibrio* (Proteobacteria) (Figure 2 and Figure 4). The comparison of taxa proportions among experimental groups identified a number of them that could have contributed to the differences observed in the microbial community (Figure 4). Statistically significant differences at the genus level were observed in the population of *Lactobacillus*, two different groups of Lachnospiraceae and *Eubacterium xylonophilum* within the class Clostridia, (*p* < 0.05) and *Barnesiella* (*p* < 0.01) within the family Porphyromonadaceae (Bacteroidetes) (Figure 4b,c). In summary, these data show significant shifts in the microbiota under inflammatory conditions induced by DSS and evident restoration effect of FMT treatment on microbial composition, which could contribute to the resolution of inflammation.

### 2.4. Colonoscopy

**Figure 5:**
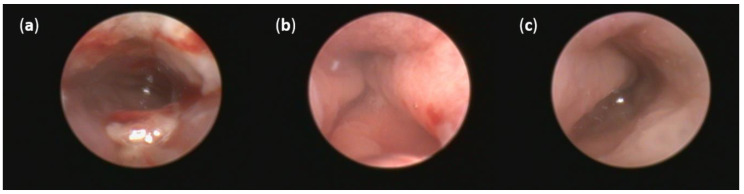
Representative pictures of colonoscopy: (**a**) Intestinal mucosa after DSS administration with the presence of many sites of mucosal reddening, profound mucosal bleeding and large fibrin deposits in the lumen; (**b**) intestinal mucosa after FMT administration with the presence of mucosal reddening and elimination of bleeding; (**c**) no thickening of the intestinal wall, bleeding or redness were observed in the group of healthy animals.

No thickening of the intestinal wall, the presence of fibrin, bleeding or redness were observed in the group of healthy animals (Figure 5c). On the other hand, oral DSS administration for seven days resulted in acute colitis in Sprague Dawley (SD) rats. Even though profound numerous sites of mucosal redness were identified in the mucosa of both DSS groups (Figure 5a,b), FMT treatment led to a decrease in fibrin deposits, elimination of spontaneous mucosal bleeding and reversed intestinal wall thickening. Moreover, colonoscopic examination revealed less mucosal inflammation in the FMT group compared with the DSS group that was associated with smaller areas of ulceration. These changes did not reach significance in final endoscopic score between DSS and FMT groups (Figure S1), but obvious trends in the improvement of clinical symptoms and the reduction of disease signs were observed in FMT group, suggesting positive effect of FMT treatment.

### 2.5. Histology and Evaluation of the Colon Length

**Figure 6:**
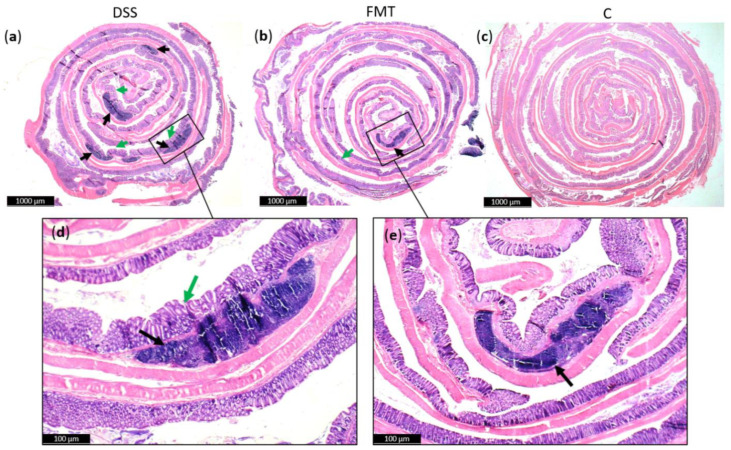
Histopathological changes in colons of experimental groups: (**a**) Numerous lymphoid follicles (black arrow) and aberrant crypts (green arrow) in the colons of acute DSS-induced colitis; (**b**) reduced damage in colon of rats treated with FMT; (**c**) no histopathological changes in colon of healthy animals; (**d**,**e**) zoom areas of colonic damage. Figure (**a**–**c**) magnification 6x; figure (**d**,**e**) magnification 16x.

**Figure 7:**
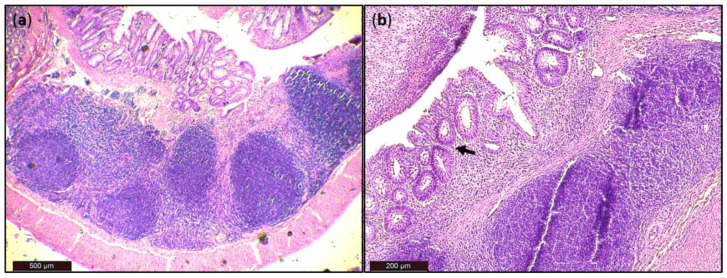
Histological damage of the colon after DSS administration. Colon tissue samples were examined using H&E staining. The colon shows signs of acute colitis: (**a**) Lymphoid follicles; (**b**) aberrant crypts—arrow.

Generally, DSS acute colitis was characterised by focal crypt lesions, goblet cell loss, diffuse lymphocytic infiltration as well as leukocyte infiltration at the areas of lesions, architectural derangements and epithelial necrosis. DSS administration caused significant observable macroscopic changes in colonic tissue architecture and tended to accelerate initial damage to the mucosa, characterised by crypt abscesses, but in some areas the inflammation resulted in extensive oedema also in the submucosa. Increased loss of goblet cells and the occurrence of more diffuse crypt architecture were detected in the DSS group compared with the FMT group, while no such lesions were observed in the colon of healthy rats (Figure 6). Numerous lymphoid follicles and crypt damage with the presence of aberrant crypts were observed in the DSS group (Figure 6a,d; Figure 7). These lesions were notably reduced by FMT treatment (Figure 6b,e). Additionally, the accumulation of leukocytes infiltrating the lamina propria could be observed more frequently in colon samples of DSS-treated rats.

**Figure 8:**
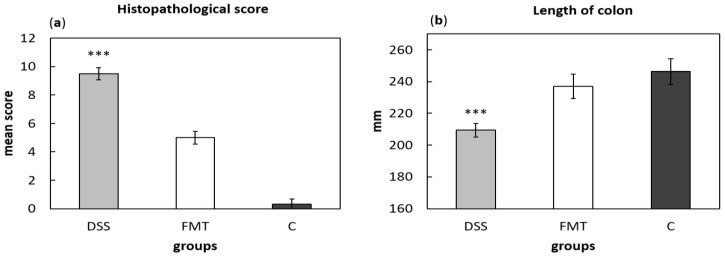
Histopathological score (**a**) and colon length (**b**) were influenced by DSS administration. Data are expressed as mean ± SEM (*** *p* < 0.001 vs. all other groups). DSS—dextran sulfate sodium induced colitis (positive control); FMT—DSS-induced colitis with subsequent FMT treatment; C—healthy control.

For histological grading of colitis severity, a histopathological scoring system identifying epithelial damage as well as inflammatory cell infiltration was used (Figure 8a). In addition, the numbers of lymphoid aggregates and follicles in histological sections were detected (Table 3). No histological damage was observed in the colon of control rats. The crypts were straight, the base of the tubular glands reached the muscularis mucosae and the epithelial cell layer on the surface of the mucosa was intact. The colon samples of DSS group, on the other hand, showed destruction of crypts and infiltration of inflammatory cells, while only partial crypt distortion and minimal infiltration of inflammatory cells were observed in FMT group. Final histopathological score was significantly reduced by FMT administration (*p* < 0.001), suggesting that FMT exerted therapeutic effects on experimental colitis. Even though no differences were observed in mean size (length and width) of individual aggregates between DSS and FMT groups, numbers of lymphoid aggregates as well as follicles per animals were significantly increased in DSS group (*p* < 0.001), suggesting lower inflammatory response (Table 3). Colon shrinkage is the indicator of UC and inflammation and as was shown in the presented study, DSS administration significantly decreased colon length in DSS group compared to healthy as well as FMT-treated animals (*p* < 0.001) (Figure 8b). Therefore, it can be concluded that FMT protected animals from colon shrinkage that was possibly associated with reduced inflammatory responses.

### 2.6. Blood Analysis

The haematological parameters of the experimental groups are represented in Table 4. Generally, the animals in DSS group showed signs of anaemia indicating loss of blood with a significant reduction in red blood cell (RBC) count (*p* < 0.001 compared to all other experimental groups) and a trend in decreasing level of haemoglobin (HGB). Moreover, platelet (PLT) count in DSS group was elevated suggesting inflammatory response and this change almost reached significance (*p* = 0.053). At the same time, slight increase in neutrophil count was also observed in DSS group, which is known to be elevated in the active stage of UC. These results suggest that FMT treatment was able to protect animals from blood loss proven by stabilization of RBC count and HGB at the level of healthy control.

**Figure 9:**
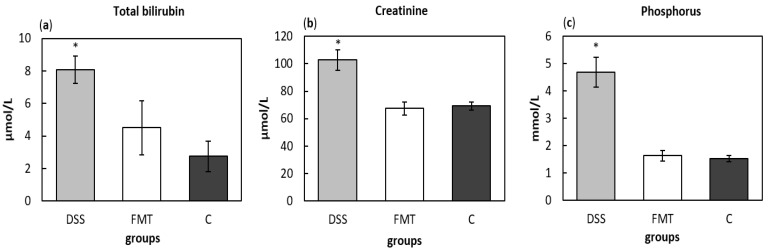
Biochemical analysis of total bilirubin (**a**), creatinine (**b**) and phosphorus (**c**) in serum samples. Data are expressed as mean ± SEM (* *p* < 0.05 vs. all other groups). DSS—dextran sulfate sodium induced colitis (positive control); FMT—DSS-induced colitis with subsequent FMT treatment; C—healthy control.

The total bilirubin levels in serum were measured to evaluate liver function and potential damaging effect of DSS, which were significantly increased (*p* < 0.05) in DSS group (8.071 ± 0.855 μmol/L), compared to healthy control (2.74 ± 0.953 μmol/L) (Figure 9a). The concentration of total bilirubin (4.5 ± 1.68 μmol/L) after FMT administration did not significantly differ from control group suggesting protective effect of faecal transplant. The determination of nitrogen profile in blood serum, namely level of creatinine as marker of renal function, showed significantly increased concentration in DSS group (102.71 ± 7.61 μmol/L), which can be sign of acute kidney injury, while no differences between FMT-treated (67.43 ± 4.70 μmol/L) and control group (69.20 ± 2.80 μmol/L) were observed (Figure 9b). The beneficial therapeutic effect of FMT was also detected in the levels of phosphorus (Figure 9c), which were significantly decreased in FMT group (1.637 ± 0.196 mmol/L) (*p* < 0.05) compared to DSS group (4.681 ± 0.545 mmol/L).

**Figure 10:**
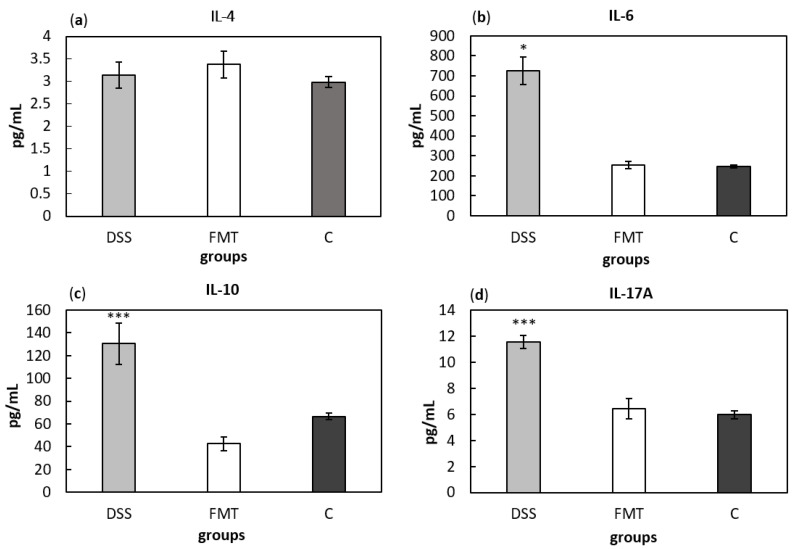
Serum levels of the cytokines IL-4 (**a**), IL-6 (**b**), IL-10 (**c**) and IL-17A (**d**). Data are expressed as mean ± SEM (* *p* < 0.05; *** *p* < 0.001 vs. all other groups). DSS—dextran sulfate sodium induced colitis (positive control); FMT—DSS-induced colitis with subsequent FMT treatment; C—healthy control.

Serum cytokine concentrations can provide information about systemic inflammatory stage of an organism. Comparing cytokine concentrations (Figure 10), significantly higher level of pro-inflammatory cytokines IL-6 and IL-17A and anti-inflammatory cytokine IL-10 were found in DSS-induced colitis group (*p* < 0.05, *p* < 0.001 and *p* < 0.001, respectively) compared to all other groups (Figure 10b–d). Finally, no differences in IL-4 concentrations were observed between experimental groups (Figure 10a). Presented results suggest decreased inflammatory response due to FMT treatment in DSS-induced colitis.

## 3. Discussion

UC is characterised by a chronic, immune-mediated intestinal inflammation affecting the innermost mucosa lining of the colon and rectum [18]. Extensive studies over the past decade have demonstrated that environmental factors, including genetic and immunological factors play a substantial role in the pathogenesis of UC [19,20]. Therefore, most of the current therapies focus on the management of the inflammation by using immune modulators. However recently, the involvement of gut microbiota is considered a new promising aspect strongly connected with UC pathogenesis [19,21], although it is still unclear whether this dysbiosis is a primary or secondary event in the relationship with gut inflammation. Nevertheless, the microbiome-based therapies are becoming a new attractive approach in the treatment of gut inflammation. The extremely high success rates achieved with FMT in the treatment of diarrhoea have catalysed the attention of researchers for its potential applications in IBD treatment.

In order to investigate the microbial associations involved in intestinal inflammation, rat models of UC are the most preferred potential systems as environmental influences and host genetics can be easily controlled. Moreover, recent study confirmed that the overall composition and structure of the faecal microbiome of rat has a greater similarity to that of the human than the microbiome of the mouse to human [22]. Furthermore, the same study also showed that the rat appeared to capture the human stool microbiome more efficiently than the mouse, highlighting the great potential of rat models to study the effect of FMT in treating IBD and other human disorders. The most widely used experimental model employs DSS to induce epithelial damage. The DSS colitis rat model has advantages over other various chemically induced experimental models due to its simplicity, reproducibility and controllability. IBD is also possible to be induced by TNBS (2,4,6-trinitrobenzene sulphonic acid), however it correlates with significant clinical and morphological features of CD and has more effectiveness in Wistar rats [23]. The inflammation induced by oral DSS administration shares similar features with UC patients from a structural, clinical and ultrastructural point of view and was shown to be more sensitive in Sprague Dawley rats [24]. Moreover, comparison at microbiome level also confirmed that the rat model of DSS-induced colitis was relatively closer to UC than to CD patients [22]. DSS mainly affects the middle and distal third of large intestine [25] and causes erosions with complete loss of surface epithelium because of its direct toxic effect on epithelial cells thus allowing bacterial infiltration into the submucosa, triggering inflammation and altering gut microbiome [26,27]. DSS-induced acute colitis is morphologically and macroscopically characterised by ulcerations, moderate to severe submucosal oedema, lesions accompanied by histopathological changes, which include infiltration of granulocytes, symptoms of which are ultimately manifested in the form of bloody diarrhoea [28]. In our study, all DSS-treated rats showed numerous clinical symptoms such as shortening of colon length, thickening of the intestinal wall, bleeding, redness of mucosa and other histological damage. Furthermore, according to recent study by Ghattamaneni et al. administration of DSS also induced selective and reversible gastrointestinal changes in rats represented by increase of Firmicutes and decrease of Bacteroidetes and Actinobacteria, providing an improved animal model taking into account bacterial dysbiosis [29].

When considering FMT as a therapy, the examination of blood and stool sample of FMT donor is very important in order to detect the presence of pathogenic species in the stool and to rule out other diseases of the donor [17]. In terms of bacterial composition, a healthy human reference microbiota list and abundance profile (Gut Feeling Knowledge Base) were recently created to provide the baseline human gut microbiota profile of healthy controls for studies related to dysbiosis. It includes all the organisms (in total 157; 8 phyla, 18 classes, 23 orders, 38 families, 59 genera and 109 species), which were confidently observed in human gut [30]. Among these, Firmicutes and Bacteroidetes dominate the gut microbiota in healthy subjects. The faecal transplant used in the present study was also comprised of eight different phyla, of which more than 80% belong to Bacteroidetes and Firmicutes. In terms of average abundance of organisms in healthy human gut [30], four phyla have abundance above 1%, these are Actinobacteria (1.82 ± 3%), Bacteroidetes (73.13 ± 22.16%), Firmicutes (22.2 ± 18.66%) and Proteobacteria (2.15 ± 10.39%) which is also in accordance with the NGS data of donor transplant in our study. Bacteroidaceae (65.58 ± 21.84%) represent the most abundant family, followed by Lachnospiraceae (11.46 ± 11.06%) and Ruminococcaceae (8.38 ± 10.48%) [30]. *Bacteroides* in the reference list came out as the most abundant genus in human gut microbiome (65.58 ± 21.84%), which includes nine species and seven of them have abundance greater than 1%. The analyses of donor faecal sample revealed similar results with *Bacteroides* confirmed as the most abundant genus in the transplant (38.05%) and species from this genus formed half of eight top species (of total 681) identified. However, one of the top species in the transplant was *Sutterella wadsworthensis,* the role of which in human gastrointestinal diseases has been suspected in the past but its specific involvement in IBD has never been firmly established [31,32,33]. However, comprehensive evaluation of colonic mucosal isolates of *S. wadsworthensis* showed no phenotypic, genotypic, proteomic or pathogenic characteristic to distinguish between bacteria isolated from patients with IBD and healthy controls [32]. Hiippala et al. showed that *Sutterella* spp. are abundant in the duodenum of healthy adults with a decreasing gradient toward the colon without any harmful effect on the enterocyte monolayer integrity in vitro [34]. The ability of *Sutterella* spp. to adhere to intestinal epithelial cells indicates that they may have an immunomodulatory role. However, cytokine response after monocyte challenge with strains from IBD patients and controls did not yield any significant differences, which indicates that *S. wadsworthensis* is unlikely to play a role in the pathogenesis of IBD and that it acts as a harmless commensal. As the donor of faecal transplant in the present study met all the requirements [35] and the examined samples had the bacterial composition of healthy human gut without detecting any pathogenic species, we concluded that donor was a suitable candidate for transplant preparation according to a standard protocol [36].

Dysbiosis is often defined as an imbalance in the complex commensal communities that is associated with specific disease. This imbalance could be due to the gain or loss of community members as well as changes in relative abundance of microbes [37]. Our results demonstrated that a healthy stool transfer for five consecutive days compensated faecal dysbiosis induced by DSS, as the relative abundance of health-related genera was increased in FMT-treated rats. A basal rat faecal microbiota content in healthy (C) rats showed that Bacteroidetes (46%) and Firmicutes (43%) represent the dominant phyla, which are complemented by Proteobacteria, Verrucomicrobia and Actinobacteria. Specific gut microbiota signatures have been detected in patients suffering from UC, however there is not a clear consensus proportion of the genus *Bacteroides* within the phylum Bacteroidetes. While some studies in the literature reported significant reduction in *Bacteroides* [38], other studies found that this group is increased in UC patients [39] and directly associated with the degradation of acidic mucin as a carbon source in the colon and with colitis [40]. Administration of DSS in our study decreased abundance of Bacteroidetes and significantly increased the population of Firmicutes. Changes in the Firmicutes-to-Bacteroidetes ratio are frequently used in the literature to indicate dysbiosis, possibly caused by disease state [41,42] and possibly due to their positive association with TNF-α and IL-1β production, which are critical cytokines involved in the regulation of immune cells [43,44,45]. In a recent study with DSS-induced colitis in mice, the microbial analysis indicated similar results with a large shift in microbiota favouring the presence of Firmicutes and suppressing Bacteroidetes due to decreased levels of unclassified genus from Bacteroidetes S24–7 family, *Bacteroides* and *Prevotella* [46]. The phylum Firmicutes includes Gram-positive bacteria with rigid or semi-rigid cell walls that are predominantly from the genera *Clostridium*, *Bacillus*, *Enterococcus*, *Lactobacillus* and *Ruminicoccus*. Despite that lactobacilli are considered as beneficial species in terms of gut protection, we showed significant decrease in their abundance after FMT treatment, which is in accordance with the study conducted by Tian et al. where the levels of lactobacilli were also significantly reduced in mice treated with FMT compared to mice treated with only DSS [47]. On the other hand, level of *Bifidobacterium* in the same study as well as in the study of Burello et al. decreased significantly in severe inflammatory phase, but it was recovered upon FMT treatment [48]. Our results showed similar trend in terms of Bifidobacteria, which are immunomodulatory short chain fatty acids (SCFA)-producing bacteria, previously shown to be associated with healthy gut ecosystems [49]. The increased levels of lactobacilli were also observed in active IBD patients, while the population of butyric acid-producing bacteria decreased to some extent [50]. Within Firmicutes, DSS administration caused evident increase in Clostridia, which was also confirmed in a mouse model of colitis conducted by Okayasu et al. [51]. Recent clinical study confirmed that other Firmicutes genera (including Lachnospiraceae, Clostridia, *Ruminococcus* genus of the Ruminococcaceae family) are increased in UC compared with CD [52]. The evidence from different studies shows that Lachnospiraceae might influence healthy functions, although different genera and species of this family are increased in diseases including IBD [53,54]. Porphyromonadaceae and Prevotellaceae families (Bacteroidetes) are also known for their association with intestinal inflammation. Porphyromonadaceae were significantly decreased in colitic mice suggesting their protective role [55], which was also supported by another study [56] where a reduction of this family in the mouse intestine contributed to severity of colitis. FMT treatment in our study helped to restore an abundance of *Barnesiella* (Porphyromonadaceae) compared to DSS group of rats. The family Prevotellaceae produces sulfatases that actively degrade mucus oligosaccharides, and as a result, might disrupt the mucosal barrier in the intestine. Study conducted by Brinkman et al. [57] suggested a possible link between the presence of this bacterial family and the severity of DSS-induced colitis. The genera *Lactobacillus* as well as some specific Clostridia, *Desulfovibrio* and *Alloprevotella* were enriched in the faecal samples of patients with chronic systemic inflammation [58]. Faecal samples of DSS rats in our study were also enriched by mentioned bacterial taxa, however FMT treatment was able to rebalance them back to the level of control rats. Although one of the top species in the transplant was *Sutterella wadsworthensis*, surprisingly the shift towards higher abundance of the genus *Suterella* was observed in DSS group of rats. Recent reports link *Sutterella* with gastrointestinal diseases, the most intriguing being therapeutic failure in UC [59]. *Sutterella* does not appear to induce substantial inflammation, rather it has a capacity to degrade IgA. Low levels of *Sutterella* within the gut microbiome are associated with gut immune homeostasis and high levels of IgA, which protects against bacterial invasion of epithelial cells by opportunistic pathobionts. Balancing the intestinal ecosystem is an important aspect of maintaining normal body function and as the administration of FMT helped to rebalance not only Firmicutes/Bacteroidetes ratio but also other less abundant bacterial populations associated with colitis, and it can represent promising therapeutic strategy to achieve/restore normobiosis during inflammation.

Colonoscopy plays a fundamental role in the diagnosis and the monitoring of disease activity [60]. As presented study showed, FMT was able to alleviate symptoms of UC, such as thickening of the intestinal wall, bleeding and presence of fibrin fibres in the intestinal lumen. Colonoscopy examination revealed mainly mucosal reddening areas in FMT-treated animals that are one of the first symptoms of IBD and according to Mayo endoscopic score (MES), such lesions are defined as MES 1 [61]. MES ≤ 1 is associated with remission stage and current therapies are primarily focused on maintaining this stage of disease for as long period of time as possible as there are no other cure available [62]. Moreover, colon shortening is always found in UC, which can act as an indirect marker of colonic inflammation [63]. Positive effect of FMT administration was also observed in regards to colon length, where this treatment was able to rescue colon shrinkage caused by DSS, which is in agreement with previously published reports [47,49,64].

Histological/ultrastructural changes of the colon in DSS-induced colitis is represented mainly by the presence of damaged goblet cell architecture in the mucosa, a significant infiltration of inflammatory cells into the stroma as well as destruction of the intestinal gland structure. Moreover, the tissue in the submucosa very often shows significant signs of oedema and hyperplasia [65]. In terms of immune cells, the infiltration of neutrophils and macrophages is observed as early as on the first day after chemical stimulation, and it is increased over time [66]. In our study, mucosal changes of the large intestine involving the rectum were localised especially in the distal colon with decreasing severity towards the proximal part. Histological examination of colonic sections revealed an altered architecture of colon mucosa with typical inflammatory changes in DSS group [65] whereas faecal transplant had protective effects and was able to restore histopathological changes in the colon possibly due to rebalanced intestinal microbiota. Similar results were obtained in human studies where FMT improved the scores for diarrhoea and mucous membrane lesions in patients with UC [67,68]. More disrupted organization of colon tissue associated with possible increased permeability of the intestinal mucosa suggests higher risk of bacterial translocation [69]. These findings were confirmed by the elevation of circulatory pro-inflammatory cytokines, which suggested that inflammatory responses in the intestine affected the changes in systemic immunity. Moreover, during inflammation the gut epithelial cells increase production of defensins, which are endogenous antibiotics with microbicidal activity against a wide range of microbes [70]. These increased small peptides may further exert their antimicrobial activity toward intestinal microbiota and thus contribute to observed dysbiosis. In mouse study, FMT treatment was able to normalize levels of defensins due to quick recovery of intestinal microbiota, better reconstruction of mucosal barriers and re-establishment of immune networks [71].

Even though no blood tests are available for IBD diagnosis, various blood parameters (e.g., leukocyte and platelet counts) are indicative of extensive, active intestinal inflammation [72]. In the presented study, DSS administration led to changes in both haematological and biochemical parameters. The most profound effects of DSS were detected on decrease in HGB level and RBC count. Such observations might be associated with massive damage of intestinal wall in untreated colitis group that resulted in blood loss. It is well known that IBD is associated with anaemia [72], therefore it can be concluded that FMT protected animals from rectal bleeding confirmed also by colonoscopic/histopathological analyses. Moreover, trend in increase of neutrophil and platelet count was obvious in the untreated group. It is well known that platelets can increase inflammatory responses through the release of many biologically active substances [73,74], whereas neutrophils are considered as the effector cells in acute and chronic inflammation with detrimental role in IBD [75]. In the mouse model of DSS-induced colitis, a reduction in neutrophils frequency and partially in absolute numbers was observed following FMT treatment [49]. Even though no statistically significant effects were observed on these cell counts, it can be assumed that FMT treatment decreased an inflammatory response in colitis group. Negative effect of DSS administration was also observed on the liver function. In healthy stage, bilirubin that arises from the breakup of haemoglobin present in red blood cells is removed by liver and excreted through bile [76]. Elevated level of bilirubin in the DSS group might be associated with liver damage induced by this chemical itself [77] or by disorders of the hepatobiliary system that are relatively common extraintestinal manifestations of IBD [78]. On the other hand, restoration of total bilirubin levels after FMT treatment at the level of healthy animals indicates a recovery of the normal liver function [79].

Clinical and epidemiological evidence suggest that IBD affects also other organs including the kidneys. Renal manifestations and complications in patients with IBD are not rare, and numerous clinical studies have reported that 4–23% of IBD patients experience renal disease [80]. Recent studies have reported that mice with colitis induced by DSS show also renal tubular injury [81,82], suggesting that this type of animal models can provide additional information on the effect of specific treatment on IBD-associated renal disease. Water-soluble DSS induces renal structural changes, particularly to the glomerular structure [83]. Creatinine has been found to be a fairly reliable indicator of kidney function [84,85] as its increased concentration in blood serum signifies possible renal malfunction. Rats in DSS group showed a significant increase in creatinine concentration compared to the healthy control and FMT-treated groups, suggesting systemic restoration effect of FMT treatment. As renal function decreases, waste products (uremic toxins) accumulate in the blood, and also increase in the intestinal epithelium [86]. This change in the gut promotes the colonization of bacteria that can use urea as an energy source by producing the enzyme urease, which catalyses the hydrolysis of urea to ammonia and carbon dioxide. Consequently, the markedly increased formation of ammonia increases the gut pH, mediates the breakdown of gut epithelial tight junctions, and facilitates endotoxemia and systemic inflammation [87]. This alteration of gut microbiota can promote the acceleration of dysbiosis and lead to the change of gut structure, including reduction of villous height, elongation of the crypts and infiltration of the lamina propria with inflammatory cells in the intestines [88]. These pathological changes in the gut suggest that translocation of bacteria and the influx of endotoxins across the intestinal wall can contribute to inducing systemic inflammation. Finally, the renal dysfunction was also confirmed by significant increase of phosphorus in serum of DSS group. Hyperphosphatemia is associated with significant kidney pathophysiology [89]. Phosphorus as the second most abundant element in the human body after calcium [90] is a component of many organic compounds and cell structures such as phospholipid cell membranes, nucleic acids and phosphoproteins [91]. Given the importance of phosphate for varied and multiple biological processes, it is not surprising that phosphate homeostasis is extremely important. Under normal physiological condition, in healthy individuals, phosphate is freely filtered through glomerulus, however its structure is changed during DSS-induced colitis, manifested by increased phosphorus levels in untreated colitic rats. In summary, our biochemical results suggest that FMT can provide another benefit to IBD patients that is the restored imbalance of gut–kidney symbiosis.

The pathogenesis of UC is related to immune dysfunction that affects mechanisms of tolerance and the innate and the adaptive immune response. It results in a dysregulation of the production of several cytokines, which play crucial roles in colitis via initiating, mediating, perpetuating and controlling intestinal inflammation. Manipulation of the gut microbiota by FMT induces variations in both innate and adaptive immune cell frequencies and cytokine profiles, which correlates with amelioration of the inflammatory status in colitic animals [48,49]. Cytokine IL-4 represents a stimulatory molecule for both B and T cells, and it is produced mainly by activated lymphocytes. Its main role stands in the inhibition of the formation of macrophage population as well as production of inflammatory mediators such as IL-1β and TNF-α [92]. The role of this cytokine in the pathogenesis of IBD is still controversial [93]. The levels of IL-4 and IL-4 mRNA were reduced in IBD, demonstrating the loss of balance between anti- and pro-inflammatory cytokines in favour of the pro-inflammatory ones [92]. Its anti-inflammatory effect was also demonstrated in patients with active UC [94]. Presented results, however, did not confirm statistically significant decrease of IL-4 in the DSS group, which is in accordance with the study conducted by Egger et al. [95]. It could be explained by the fact that unlike in human disease, T and B cells are not required for development of colitis induced by DSS [96]. Hence, the acute DSS colitis model is particularly useful when studying the contribution of the innate immune system to the development of intestinal inflammation. This apparent limitation of DSS model is the reason why we could not obtain any significant results related to cytokine IL-4. Another anti-inflammatory cytokine tested in presented study was IL-10 that was shown to have a protective role within the mucosal immune system. This cytokine have been studied very extensively in UC [97] and it was confirmed that inactivation of the gene for IL-10 in mice causes a chronic ileocolitis [98] and that its concentration was significantly elevated in UC patients with aim to decrease inflammatory reactions in the gut [99,100]. Therefore, increased levels of IL-10 in untreated DSS rats in our study could be associated with an attempt of the immune system to control inflammation in rat colon, suggesting more severe disease course [99,100]. Similar results were observed by the study Egger et al. where significant upregulation of IL-10 expression was associated with increasing severity of the colonic injury [95]. Different animal models have shown that reduced colonic inflammation following FMT was due to increase in IL-10 production [49,64,97,101], which is in contrast with our results. However, based on our histological/microbial/haematological analyses we can assume that FMT treatment was effective in fostering resolution of inflammation manifested by decreased levels of IL-10. Similar observation was reported by Burrello et al. where increased IL-10 secretion by T cells observed in FMT-treated mice was normalised upon inflammation resolution [49]. Cytokine IL-6 is a typical pro-inflammatory cytokine, the production of which is induced during the acute-phase response as it triggers anti-apoptotic signals in T cells and stimulates production of inflammatory cytokines in many cell types [102]. In UC patients, elevated IL-6 levels were found in serum [103,104,105] and were correlated with disease severity [105,106]. The serum level of IL-6 was significantly positively correlated with CRP, as well as with Mayo score [107]. Experiment in mice showed that the tissue level of IL-6 was down-regulated after FMT treatment, and reduced even more during the remission phase [47], which is in accordance with our study. Wang et al. suggested that the level of IL-6 can represent objective indicator for the responsiveness of UC patients to FMT treatment [107]. Finally, IL-17A is a pro-inflammatory cytokine, which is produced mainly by Th17 cells associated with immune-mediated diseases. Initial studies have shown increased IL-17A mRNA expression and increased numbers of Th17 cells in the inflamed tissue compared to healthy mucosa [108,109]. Furthermore, IL-17A induces the recruitment and activation of granulocytes and locally promotes the production of other pro-inflammatory cytokines such as TNF-α, IL-6 and IL-1β [110,111]. Our results clearly demonstrated, that FMT therapy alleviated the intestinal inflammation, as confirmed by a robust decline of main pro-inflammatory cytokines associated with UC. These results are in accordance with a previous report [48].

## 4. Materials and Methods

### 4.1. Animals

Male SD (*n* = 18) rats with conventional microbiological status (VELAZ, Prague, Czech Republic) were obtained at six weeks of age, quarantined and housed in groups of four per cage at room temperature 22 ± 2 °C, 55 ± 10% humidity and a 12 h light/dark cycle. They had free access to a standard laboratory diet (Altromin 1324, Lage, Germany) and deionized water. Rats were divided into 3 groups, namely the healthy control (C) (*n* = 6), DSS (*n* = 6) and FMT group (*n* = 6). Acute experimental colitis was induced in DSS and FMT groups using DSS (40 kD; TdB Consultancy AB, Uppsala, Sweden), as previously described [112]. Briefly, the rats were provided with deionized water containing 5% DSS for 7 days. On day 8, the FMT group was treated with orally administered (200 μL) human FMT once of the day for additional 5 days and its therapeutic effect was evaluated. Food, DSS/water consumption and the body weight were measured on a daily basis. All rats were sacrificed on day 13 by rapid decapitation. The presented experiment was approved by the National Animal Ethical Committee of the Slovak Republic (license number Ro-1222-3/2020-220) and was conducted in accordance with the European Convention for the protection of vertebrate animals used for experimental and other scientific purposes (ETS 123).

### 4.2. Faecal Transplant

Donors of faecal transplant with neither symptoms nor a family history of gastrointestinal disorder and with no use of antibiotics within the preceding six months participated in the study. Donors underwent screening tests of blood and stool before the donation according to recommendations [113]. Fully screened [114] healthy donors provided at least 50 g of fresh stool, which was placed immediately in anaerobic conditions using a catalyst (GENbag Anaert; Biomérieux SA, Marcy l’Etoile, France) according to the International Human Microbiome Standards [115]. Faecal transplant was then prepared using previously described experimental protocol [36]. Briefly, stool specimen was mixed with 250 mL (1:6 dilution) sterilised 0.9% NaCl using sterile stainless-steel homogenizer (Waring 7011HS Speed Heavy-Duty Lab Blender, Montreal, Canada) until a smooth consistency was reached. The final product was filtered through 2.0, 1.0, 0.5 and 0.25 mm stainless-steel sieves and the resulting material was centrifuged at 4 °C, 6000× *g* for 15 min. The pellet was resuspended with 125 ml sterile of 0.9% NaCl and glycerol was added up to the final concentration of 10%. The final suspension was aliquoted into sterile individual cryotolerant tubes and stored at −80 °C. Prior to use the faecal transplant was thawed in a warm (37 °C) water bath and was orally administered to the rats in FMT group.

### 4.3. Microbiome Analysis of Transplant and Rat Faecal Samples

Faecal samples were collected at the end of experiment and stored at −80 °C until DNA extraction. The stool specimens were then thawed gently on ice, homogenised by manual mixing and weighed according to the recommendations of NucleoSpin^®^ DNA Stool kit (Machery-Nagel, Düren, Germany). The extractions were performed according to the manufacturers’ instructions. The DNA concentrations of the extracts were measured fluorometrically with the Qubit dsDNA HS assay kit (Thermo Fisher Scientific, Waltham, USA), after which the DNA samples were stored at −20 °C until 16S rDNA library preparation. Faecal DNA samples together with FMT sample were analysed by commercially available NGS service (Novogene Europe, Cambridge, UK). Briefly, hypervariable regions of V3-V4 in bacterial 16S rDNA were amplified using primers 341F (CCTAYGGGRBGCASCAG) and 806R (GGACTACNNGGGTATCTAAT) and subjected to subsequent taxonomic analysis. All PCR reactions were carried out with Phusion^®^ High-Fidelity PCR Master Mix (New England Biolabs, Ipswich, MA, USA). The libraries were generated with NEBNext^®^ Ultra^TM^ (New England Biolabs, Ipswich, MA, USA) DNA Library Prep Kit for Illumina, quantified via Qubit 2.0 Fluorometer and sequenced using Illumina Novaseq PE250. The statistical analyses using QIIME, Flash, R, RDP, Blast (ITS), PyNast bioinformatics software were performed by Novogene Europe (Cambridge, UK).

### 4.4. Colonoscopic Examination and Scoring

Colonoscopy with photo documentation was performed at the end of experiment. Prior to endoscopic visualisation, animals were fasted overnight. Rats were anaesthetised by isoflurane inhalation (RWD Life Sciences, San Diego, CA, USA) and placed in a supine position. Before the insertion of rigid endoscope Tele Pack Vet X LED RP 100 (Karl Storz, Tuttlingen, Germany), any remaining stool was removed by massaging the anus. The endoscope was inserted 10 cm proximally into the anus. A modified Endoscopic Index of Colitis Severity [116] (EICS, 0–12) was used for endoscopic evaluation and grading of the colitis. Epithelium of the colon was examined in 4 categories (Table 5): Vascular translucency, presence of fibrin deposits, bleeding and reddening of the colon mucosa. The examination was done in a blinded manner and the final score was calculated as the sum of each examined categories.

### 4.5. Histology

After decapitation, the entire colon was removed and its length was measured. The colon was opened longitudinally, processed by the Swiss roll method [117] and fixed in formalin. For histological observation, the tissue was dehydrated, embedded in paraffin blocks, sectioned (5 µm), stained with H&E and scored by two pathologists “blinded” to sample identity. The microscopic colonic epithelial damage was scored as: 0 = normal; 1 = hyperproliferation, irregular crypts, and goblet cell loss; 2 = mild to moderate crypt loss (10–50%); 3 = severe crypt loss (50–90%); 4 = complete crypt loss, surface epithelium intact; 5 = small-to medium-sized ulcer (<10 crypt widths); and 6 = large ulcer (≥10 crypt widths) [118]. Infiltration with inflammatory cells was scored separately: Mucosa (0 = normal, 1 = mild, 2 = modest, 3 = severe), submucosa (0 = normal, 1 = mild to modest, 2 = severe), and muscle/serosa (0 = normal, 1 = moderate to severe). Scores for epithelial damage and inflammatory cell infiltration depending on colonic depth were added, resulting in a total scoring range of 0 to 12. The number and size of lymphoid aggregates were also measured by the light microscope Leica DM2500 (Leica Microsystems, Wetzlar, Germany).

### 4.6. Blood Analysis

Blood samples were obtained during decapitation. Two hundred microlitres of blood was collected into K_3_EDTA-containing tubes and 800 µL into serum gel tubes (Sarstedt, Nümbrecht, Germany). K_3_EDTA blood samples were analysed using an automatic analyser (EXIGO H400, Boule Medical AB, Spånga, Sweden). Blood for serum collection was allowed to clot for at least 30 min and subsequently tubes were centrifuged at 2400× *g* at 4 °C for 15 min. Serum samples were stored at −20 °C and biochemical analyses were performed over 2 consecutive days using an automated clinical chemistry analyser (ELLIPSE; AMS SpA, Rome, Italy) according to the manufacturer’s instructions. To measure the serum concentrations of IL-4, IL-6, IL-10 and IL-17A, an enzyme-linked immunosorbent assay (ELISA) was performed using commercially available kits for cytokine detection (IL-4, IL-6 and IL-10 (eBioscience, San Diego, CA, USA); IL-17A (BlueGene, Shanghai, China) and applying the manufacturer’s recommended procedure.

### 4.7. Statistical Analysis

All statistical analyses were performed using Minitab software version 16 (Minitab Inc., 2013, State College, PA, USA). All data were first tested for normal distribution and then appropriate tests were used. Statistical differences between groups were analysed using ANOVA test, followed by Tukey post hoc test or Kruskal–Wallis test followed by pairwise multiple comparison procedures (Mann–Whitney test). All differences were considered statistically significant at a significance level of *p* < 0.05.

## 5. Conclusions

Our study demonstrated significant shifts in the dominant species of microbiota under inflammatory conditions induced by DSS and evident restorative effect of FMT treatment on microbial composition. Because the dominant microbiota exerts the major effects on the gut immune system and epithelial barrier function, FMT thus provides very promising strategy to the resolution of inflammation during acute colitis. Even without knowing the functions of every species in gut microbiota, it is possible to use FMT as a therapeutic approach. FMT-treated animals exhibited reduced disease severity, with significantly less colonic injury, including reduced colon ulceration, crypt damage and inflammation. However, future studies are necessary to determine whether personalised faecal transplant prepared by its enrichment with a single or mixture of probiotic species specifically selected on the basis of the patient′s immune status, microbial composition and type/state of disease will provide more efficient treatment of the gut inflammatory diseases. If FMT proves to be effective in the management of UC, another question remains to be answered and that is which component of FMT is responsible for its therapeutic efficiency. In fact, it is very important to test/explain the mechanisms that underpin successful FMT for specific disease indication as this may allow us to refine FMT into a more targeted, efficacious and safer therapy.

## Figures and Tables

**Table 1:** Effect of dextran sulfate sodium (DSS) and faecal microbiota transplant (FMT) on body weight gain and food intake.

Groups	Body Weight Gain (g)	Food Intake (g/rat/day)	FER ^1^ (%)
DSS	55.2 ± 5.19	30.94 ± 0.76 ^A^	15.16 ± 1.37 ^a^
FMT	66.0 ± 1.79	27.60 ± 0.66 ^B^	20.08 ± 1.05 ^b^
C	62.0 ± 1.47	28.48 ± 0.40 ^B^	18.17 ± 0.90 ^ab^

Values are expressed as mean ± SEM. Means with the same superscript are not significantly different; different superscript letters indicate significant difference, where *p* < 0.05 (a, b) and *p* < 0.001 (A, B). ^1^ FER (food efficiency ratio) (%) = (body-weight gain/food intake) ×100. DSS—dextran sulfate sodium induced colitis (positive control); FMT—DSS-induced colitis with subsequent FMT treatment; C—healthy control.

**Table 2:** Top 8 of 32 phylum classification results of human transplant.

Classification	Number of Reads	% Total Reads
Bacteroidetes	124,394	58.93
Firmicutes	50,658	24.00
Proteobacteria	23,739	11.25
Actinobacteria	10,624	5.03
Unclassified	1193	0.57
Armatimonadetes	189	0.09
Acidobacteria	98	0.05
Candidatus_Saccharibacteria	42	0.02

**Table 3:** Evaluation of the lymphoid aggregates.

Group	LA per Animal	LF per Animal	Mean Length of LA (μm)	Mean Width of LA (μm)
DSS	3.33 ± 0.21 ^A^	15.5 ± 0.89 ^A^	1881 ± 147 ^A^	445 ± 26 ^A^
FMT	2.17 ± 0.17 ^B^	8 ± 0.58 ^B^	1388 ± 239 ^A^	412 ± 49 ^A^
C	0.5 ± 0.22 ^C^	0.67 ± 0.33 ^C^	333 ± 179 ^B^	95 ± 56 ^B^

All values in the table are expressed as mean ± SEM (*n* = 6/experimental group). Different superscript letters (A, B, C) indicate significant difference (*p* < 0.001). Abbreviations: LA—lymphoid aggregates; LF—lymphoid follicles. DSS—dextran sulfate sodium induced colitis (positive control); FMT—DSS-induced colitis with subsequent FMT treatment; C—healthy control.

**Table 4:** White and red blood cell count values.

	DSS	FMT	C
WBC (10^9^/L)	8.425 ± 0.297	9.160 ± 0.705	8.362 ± 0.572
LYM (10^9^/L)	6.425 ± 0.254	7.060 ± 0.574	6.675 ± 0.458
MON (10^9^/L)	0.383 ± 0.0423	0.390 ± 0.050	0.313 ± 0.040
GRA (10^9^/L)	1.716 ± 0.065	1.780 ± 0.146	1.475 ± 0.246
NEU (10^9^/L)	1.367 ± 0.131	1.260 ± 0.121	1.175 ± 0.125
EOS (10^9^/L)	0.200 ± 0.058	0.420 ± 0.201	0.125 ± 0.025
HGB (g/L)	145.13 ± 0.55	150.25 ± 0.85	158.42 ± 5.24
RBC (10^12^/L)	6.783 ± 0.037 ^A^	7.058 ± 0.037 ^B^	7.125 ± 0.025 ^B^
PLT (10^9^/L)	1059.8 ± 24.1	937.0 ± 79.5	896.8 ± 18.3

All values in the table are expressed as mean ± SEM (*n* = 6/experimental group). Different superscript letters indicate significant difference (*p* < 0.001). Abbreviations: WBC—white blood cells; LYM—lymphocytes; MON—monocytes; GRA—granulocytes; NEU—neutrophils; EOS—eosinophils; HGB—haemoglobin; RBC—red blood cell; PLT—platelets. DSS—dextran sulfate sodium induced colitis (positive control); FMT—DSS-induced colitis with subsequent FMT treatment; C—healthy control.

**Table 5:** Modified endoscopic index of colitis severity (EICS) based on the observed signs of inflammation. Four parameters were examined, as indicated.

Category	Score	Description
Translucency	0	Vascularisation fully visible
	1	Vascularisation partially visible
	2	Vascularisation not visible
Fibrin	0	No fibrin is present in the lumen
	1	Small fibrin deposits in the lumen
	2	Large fibrin deposits in the lumen
Bleeding	0	No bleeding
	1	Several sites of mucosal bleeding
	2	Many sites of mucosal bleeding, may obstruct camera of the endoscope, bleeding may start spontaneously or as a reaction to contact with endoscope, blood may directly flow out of the anus
	3	Profound mucosal bleeding, usually obstructs camera of the endoscope, bleeding often starts spontaneously and blood usually flows out of the anus
Reddening	0	No reddening visible
	1	Several sites of mucosal reddening
	2	Many sites of mucosal reddening

## Supplementary Materials

The following is available online at https://www.mdpi.com/2076-0817/10/2/152/s1, Figure S1: Colonoscopic score.
