# Supplementary material for: Single Donor FMT Reverses Microbial/Immune Dysbiosis and Induces Clinical Remission in a Rat Model of Acute Colitis
Source: Pathogens. 2021 Feb 2;10(2):152. doi: 10.3390/pathogens10020152 (PMC7913212; doi:10.3390/pathogens10020152)
Supplement: Supplementary file 1 [file pathogens-10-00152-s001.pdf]

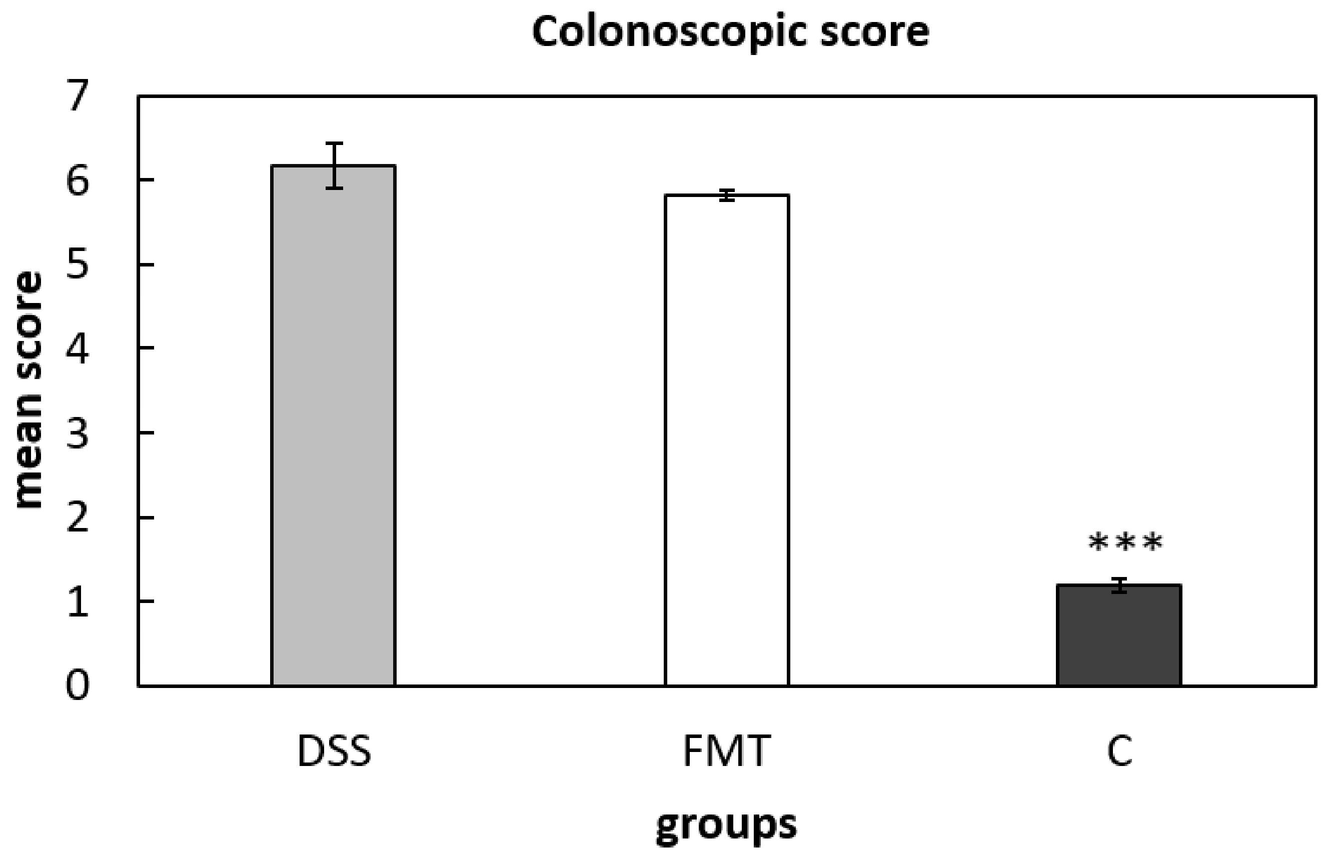

Figure S1. Colonoscopic score. Data are expressed as mean ± SEM (***p<0.001 vs. all other groups). DSS – dextran sulfate sodium induced colitis (positive control); FMT – DSS-induced colitis with subsequent FMT treatment; C – healthy control.
